# Integrating stem cell-based experiments in clinical research

**DOI:** 10.1192/j.eurpsy.2020.64

**Published:** 2020-06-15

**Authors:** Rakesh Karmacharya, Christian Kieling, Valeria Mondelli

**Affiliations:** 1 Program in Neuroscience and Chemical Biology, Center for Genomic Medicine, Massachusetts General Hospital & McLean Hospital, Harvard University, Boston, MA, USA; 2 Chemical Biology and Therapeutic Science Program, Broad Institute of Harvard & MIT, Cambridge, MA, USA; 3 Department of Psychiatry, Universidade Federal do Rio Grande do Sul, Porto Alegre, Rio Grande do Sul, Brazil; 4 Child & Adolescent Psychiatry Division, Hospital de Clínicas de Porto Alegre, Porto Alegre, Rio Grande do Sul, Brazil; 5 Department of Psychological Medicine, Institute of Psychiatry, Psychology & Neuroscience, King's College London, London, United Kingdom; 6 National Institute for Health Research Mental Health Biomedical Research Centre, South London and Maudsley NHS Foundation Trust, and King's College London, London, United Kingdom

## Abstract

With the seminal discovery of somatic cell reprogramming with defined genetic factors, it is now a routine laboratory procedure to reprogram somatic cells to generate patient-specific induced pluripotent stem cells (iPSCs) [1] Patient-specific iPSCs can be differentiated to generate mature neurons as well as three-dimensional brain organoids that show appropriate functional activity in electrophysiological studies [2,3]. However, there is a significant gap in the thoughtful incorporation of patient-derived neuronal cells in clinical studies addressing disease risk.

With the seminal discovery of somatic cell reprogramming with defined genetic factors, it is now a routine laboratory procedure to reprogram somatic cells to generate patient-specific induced pluripotent stem cells (iPSCs) [1] Patient-specific iPSCs can be differentiated to generate mature neurons as well as three-dimensional brain organoids that show appropriate functional activity in electrophysiological studies [2,3]. However, there is a significant gap in the thoughtful incorporation of patient-derived neuronal cells in clinical studies addressing disease risk.

Recently, we have seen the implementation of well-designed clinical studies aimed at delineating biological and psychosocial risk factors for psychiatric disorders. One such example is the Identifying Depression in Adolescence, a multisite global study spanning four continents that has developed an empirically derived stratification tool for the identification of adolescents at high and low risk for developing depression [4]. These studies have the potential to undertake deep phenotyping of symptoms and risks while performing incisive biological studies. While such clinical cohort studies have been undertaken in the past with serum, fibroblasts, or peripheral blood mononuclear cells (PBMCs), it behooves the clinical and translational research community to utilize patient-derived neuronal cells, given the central role of the brain in psychiatric disorders.

The ability to generate specific neuronal and glial cell types as well as three-dimensional brain organoids with disease-specific genetic backgrounds provides new opportunities for targeted biological studies [5]. For instance, given the role of microglia and neuroinflammation in psychiatric disorders [6], iPSC-derived neurons and microglia can be used to investigate glia–neuron interaction in these disease contexts. Part of the historical challenge in psychiatry has been a lack of fruitful engagement between basic scientists and clinical researchers. Basic scientists often treat diagnostic labels as monolithic entities, with little understanding of the nuances of psychiatric disorders. On the other hand, most clinical researchers are not well-versed in cellular and molecular biology techniques that can inform on mechanistic bases of disease biology. There is a need for cross-fertilization of ideas and methodologies and active collaboration between these two groups of investigators.

Human neuronal cells with disease-specific genetic backgrounds provide novel ways to explore disease biology (Figure 1). We can pursue hypothesis-based studies to test specific theories about the biology underlying disease pathophysiology/risk, that is, cell-autonomous deficits in specific neuronal subtypes in a particular disorder [7]. Another approach involves using discovery-based approaches to profile-specific neuronal cells with transcriptomic, proteomic, phospho-proteomic, and metabolomic experiments. Gene–environment interactions play a pivotal role in many psychiatric disorders. Profiling patient cells in the setting of environmental perturbations can be used to uncover biological features that may not be present under normal culture conditions [8,9]. We can investigate how specific neuronal subtypes from disease subjects may behave differently in the setting of specific environmental stressors. For instance, do neural stem cells from youth at high risk for developing depression show differences in neurogenesis or show differential regulation of specific sets of genes when exposed to stress hormones, sex hormones, or pro-inflammatory cytokines? Such studies can help identify biological pathways that underlie the clinical manifestations of psychiatric disorders.

**Figure 1.**
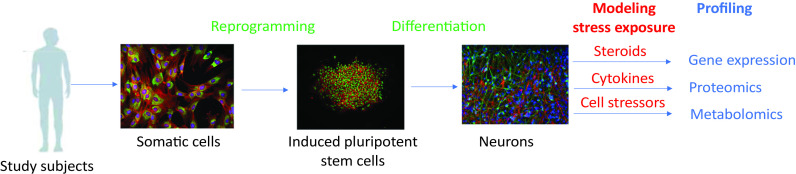
Framework for utilizing human system cell-derived neurons in clinical cohort studies.

There are subgroups of patients in the disease categories that respond to specific treatments while others do not. The differential treatment response is hypothesized to be due to different underlying biology in these subgroups [10]. It would be tractable to generate neurons from treatment responders and nonresponders and undertake *in vitro* experiments to identify mechanisms underlying differential response to treatment. Identification of cellular features that correlate with treatment response not only provides face validity to the mechanistic basis for the disease biology, but it will also aid in the development of predictive and precision medicine methods. With the identification of a robust and reliable treatment-response signature, one can evaluate effects of potential medications in neurons from individual patients before making an educated decision to pick the medication that shows the most robust response in the *in vitro* assays for that particular patient.

We are at an opportune juncture for the application of cutting-edge stem cell approaches to study psychiatric disease biology. In order to successfully bring these new technologies to the service of psychiatric research, we need a more engaged collaboration between clinical researchers and basic scientists. It will also require funding agencies and academic journals to view this rich area of potential research with new lenses and involve translational researchers who understand the new technologies and at the same time can appreciate the intricacies involved in their application to the complex issues involved in clinical psychiatry research.
